# Association of coping pathways with anxiety symptoms in children with hematological cancers: a cross-sectional study using regression and fuzzy-set qualitative comparative analysis

**DOI:** 10.1186/s40359-026-05216-8

**Published:** 2026-07-27

**Authors:** Shishuang Zhou, Can Gu, Weiwei Liu, Nan Hou, Mei Fu, Lin Mo, Cho Lee Wong

**Affiliations:** 1 The Nethersole School of Nursing, Faculty of Medicine, The Chinese University of Hong Kong, Hong Kong, China; 2 XiangYa Nursing School, Central South University, ChangSha, China; 3 School of Nursing, Army Medical University, ChongQing, China; 4 Systems Engineering and Engineering Management, The Chinese University of Hong Kong, Hong Kong, China; 5 Emergency Medical Rescue Command and Dispatch Centre of Qianxinan Prefecture, Qianxinan, Xingyi, GuiZhou Province China; 6 Department of Outpatient Children’s Hospital of Chongqing Medical University, National Clinical Research Centre for Children and Adolescents’ Health and Diseases, Ministry of Education Key Laboratory of Child Development and Disorders, International Science and Technology Cooperation Base of Child Development and Critical Disorders, Chongqing Key Laboratory of Child Neurodevelopment and Cognitive Disorders, ChongQing, China

**Keywords:** Anxiety, Coping strategies, Childhood, Haematological cancer, Cross-sectional study; fsQCA

## Abstract

**Background:**

Children with hematological cancers are vulnerable to anxiety symptoms during chemotherapy. Coping is a modifiable psychosocial factor that can be assessed and supported in pediatric oncology nursing practice. However, previous studies have mainly examined coping strategies separately, providing limited insight into how different coping characteristics may co-occur among children with varying levels of anxiety symptoms. This study aimed to examine coping-related factors associated with anxiety symptoms and to identify coping configurations associated with high and low anxiety symptoms.

**Methods:**

A cross-sectional study was conducted among 390 children aged 7–14 years with hematological cancers from four hospitals in China. Data on anxiety symptoms, coping strategies, and demographic and clinical characteristics were collected using structured questionnaires. Multiple linear regression was used to identify coping-related factors independently associated with anxiety symptoms. Fuzzy-set qualitative comparative analysis (fsQCA) was then applied to explore combinations of coping-related conditions associated with high and low anxiety symptoms.

**Results:**

Emotional reaction coping and self-comfort coping were positively associated with anxiety symptoms, whereas global coping self-assessment was negatively associated with anxiety symptoms. Male sex was associated with lower anxiety symptoms compared with female sex. The regression model explained 47.1% of the variance in anxiety symptoms. identified multiple configurations associated with high and low anxiety symptoms, suggesting that different anxiety symptom levels may be related to different combinations of coping characteristics. High anxiety symptoms were commonly characterized by maladaptive coping, limited adaptive coping resources, and reduced emotional expression. In contrast, low anxiety symptoms were associated with stronger global coping self-assessment, lower emotional reactivity, and lower avoidance.

**Conclusions:**

Anxiety symptoms among children with hematological cancers were associated with both individual coping characteristics and broader coping configurations. By integrating regression analysis with fsQCA, this study provides complementary evidence on variable-level associations and configurational patterns. The findings support routine coping assessment and profile-informed psychosocial care to help identify children with higher anxiety symptoms and guide targeted supportive interventions during chemotherapy.

**Supplementary Information:**

The online version contains supplementary material available at https://doi.org/10.1186/s40359-026-05216-8.

## Introduction

Childhood cancer remains a major global health concern. The World Health Organization (WHO) estimates that approximately 400,000 children and adolescents aged 0–19 years develop cancer each year, while hematological cancers are among the most common childhood malignancies [1]. Although the survival of pediatric cancer patients has improved substantially in settings with access to comprehensive care, diagnosis and treatment continue to impose considerable psychological burdens on children and their families [2]. Recent evidence suggests that this burden extends beyond physical symptoms to include an increased risk of anxiety symptoms and other mental health problems across the disease trajectory [3].

In children with hematological cancers, anxiety symptoms arise from the uniquely intensive and protracted nature of their treatment and are a major clinical concern [3, 4]. Pediatric oncology settings require rigorous progression through the induction, consolidation, and maintenance phases, most notably in cases of childhood acute lymphoblastic leukemia. In each of these phases, the child is subjected to frequent invasive procedures, sustained chemotherapy demands, and significant disruptions to daily life [4]. These persistent and only partly controllable stressors may be associated with increased psychological distress, with anxiety symptoms representing one important manifestation.

Coping, a key construct for understanding pediatric responses to oncological stress [5], encompasses the cognitive, emotional, and behavioral efforts employed to manage disease-related demands [6]. Recent studies have suggested that the relationship between coping and anxiety is nuanced; coping is intrinsically linked to emotional adaptation, yet its impact varies widely, depending on the specific strategies used [5, 7]. For example, acceptance and cognitive-palliative coping may help children tolerate or reinterpret treatment-related stress, whereas avoidance, distancing, wishful thinking, and emotional reaction may reflect different ways of managing overwhelming illness-related experiences. Despite these insights, the literature remains inconclusive regarding which coping patterns can most effectively mitigate anxiety symptoms in young patients. Comparative studies have identified significant gaps in our understanding of child-reported coping mechanisms and their direct associations with anxiety symptoms [5]. Furthermore, heterogeneity in disease phase, assessment approaches, and contextual factors across previous studies may limit the comparability of findings, underscoring the need for more illness-specific investigations.

This gap is especially relevant among children with hematological cancers. Much of the existing psycho-oncology literature involves broad samples of pediatric cancer patients, caregiver outcomes, survivorship, or psychosocial distress, rather than focusing specifically on anxiety symptoms in children undergoing active treatment for hematological disease [8, 9]. In parallel, psychosocial care frameworks for pediatric oncology emphasize the importance of early identification, systematic assessment, and timely intervention to address emotional distress [10]. Recent work has shown that caregivers and clinicians prioritize evidence-based psychosocial care and that the integration of psychological services at diagnosis is both feasible and valued [11]. Together, these findings suggest that the identification of modifiable psychological processes associated with anxiety symptoms is not only academically relevant but also directly meaningful for pediatric oncology practice and nursing care.

The literature is further limited by methodological considerations. Traditional research has predominantly relied on variable-centered approaches to estimate the independent associations of individual predictors [12, 13]. Although these approaches are useful for identifying salient correlations, they may not fully capture how multiple coping characteristics co-occur in relation to anxiety symptoms. In pediatric oncology settings, anxiety symptoms may be associated not only with single coping dimensions but also with broader combinations of emotional, cognitive, and behavioral coping responses. Complementing regression analysis with configurational analysis may therefore provide a more comprehensive assessment of how different coping characteristics combine in relation to anxiety symptoms. Fuzzy-set qualitative comparative analysis (fsQCA) is a configurational method designed to identify multiple pathways, or combinations of conditions, associated with a given outcome [14, 15]. FsQCA may be useful for pediatric oncology research because children with similar anxiety symptom levels may present different coping profiles. While recent reviews have highlighted the growing recognition of the value of fsQCA in research on child well-being, due to its ability to capture heterogeneity, the application of this approach to nursing research remains nascent [16].

This study used a dual-method approach to examine the associations between coping strategies and anxiety symptoms in children with hematological cancers. Multivariate linear regression identified coping dimensions independently associated with anxiety symptoms, while fsQCA explored coping-related configurations associated with high and low anxiety symptoms. Regression analysis provides evidence on variable-level associations, whereas fsQCA provides complementary insight into configurational patterns. Together, these approaches may support routine assessment of children’s coping characteristics in pediatric oncology nursing practice, facilitate earlier identification of children with higher anxiety symptom.

## Materials and methods

This cross-sectional study was reported in accordance with the Strengthening the Reporting of Observational Studies in Epidemiology (STROBE) guidelines.

### Study aim

The aim of this study was to examine the associations between coping strategies and anxiety symptoms in children with hematological cancers, and to identify coping-related configurations associated with high and low anxiety symptoms using regression analysis and fuzzy-set qualitative comparative analysis.

### Study design and participants

This cross-sectional study examined the association between coping strategies and anxiety symptoms among children with hematological cancers. Participants were recruited from four hospitals in China between February and December 2025. A convenience sampling approach was applied across the study sites.

A total of 960 children were screened, of whom 450 met the inclusion and exclusion criteria. Of these eligible children, 390 agreed to participate and completed the online questionnaire. The remaining 60 declined to participate, primarily because of lack of interest or insufficient time. The response rate among eligible children was 86.7% (390/450).

Eligible participants were children aged 7–14 years who had been diagnosed with hematological cancer within the past year, were receiving chemotherapy at the time of data collection, and were able to understand and complete the questionnaire with support from trained researchers when needed. Efforts were made to include children from different age groups and diagnostic categories to ensure variability within the sample. Participants were excluded if they had documented developmental, neurological, or psychiatric conditions or were currently involved in other research studies. Child assent and written informed consent from parents or legal guardians were obtained before participation.

The sample size was pragmatically determined by recruiting all eligible children with hematological cancers from the four hospitals during the study period. For regression analyses, this sample was sufficient to detect at least moderate effect sizes with statistical power above 80% at α = 0.05, consistent with conventional power analysis principles [17]. The sample was also considered adequate for multivariable model estimation and fsQCA, which benefits from medium-to-large samples to support calibration and configurational analysis [18].

### Data collection

Data were collected using structured self-report questionnaires administered in a clinical setting. Questionnaires were completed using the online survey platform Wenjuanxing (https://www.wjx.cn), which has been widely used in survey-based research in China. Trained researchers were present during data collection to provide clarification and ensure comprehension. The participants’ basic demographic and clinical information, including age, gender, sibling status, insurance status, and emotional expression (e.g., whether the child tends to express distress), was also collected. Incomplete or invalid responses were excluded before analysis.

### Measures

#### Anxiety symptoms

Anxiety symptoms were assessed using the Chinese version of the Spence Children’s Anxiety Scale–Short Version (SCAS-S) [19]. The scale consists of 19 items rated on a 4-point Likert scale (0 = never to 3 = always). The total scores range from 0 to 57, with higher scores indicating more severe anxiety symptoms. The Chinese version has demonstrated good internal consistenanxeicy in previous research (Cronbach’s α = 0.86) [19]. In the present study, the anxiety scale demonstrated excellent internal consistency (Cronbach’s α = 0.921).

#### Coping strategies

Coping strategies were measured using the Chinese version of the Coping with Disease Inventory (CODI) [20]. This instrument comprises 28 items in six coping dimensions: acceptance, emotional reaction, distancing, wishful thinking, self-comfort, and avoidance. The items are rated on a 5-point Likert scale ranging from 1 (never) to 5 (always). Subscale scores are calculated by summing the items for each coping dimension, with higher scores indicating greater use of the corresponding coping strategy. To remain consistent with the original scale structure, no total coping score was computed in this study, as the subscales represent distinct coping dimensions rather than a unidimensional construct. The final item (Item 28) is a single-item global coping self-assessment. In the current sample, for the coping questionnaire, Cronbach’s α ranged from 0.836 to 0.930 across the six coping subscales, indicating good internal consistency.

### Statistical analysis

All analyses were conducted in Python 3.13.11 using pandas, NumPy, SciPy, statsmodels, and scikit-learn. The analyses were based on the available cases after data cleaning. Descriptive statistics were used to summarize the sample characteristics and study variables. Continuous variables are reported here as means and standard deviations, and categorical variables as frequencies and percentages. Pearson correlation analysis was conducted to examine potential overlap among coping dimensions before regression and fsQCA analyses.

#### Regression analysis

Multiple linear regression analysis was performed to examine the associations between coping strategies and anxiety symptoms. Demographic variables, including age, gender, sibling status, insurance status, and emotional expression, were entered as control variables in the first block, followed by coping-related variables. The model fit was evaluated using the R² and adjusted R² values. Standardized regression coefficients (β) and corresponding *p*-values were reported to identify significant predictors of anxiety symptoms.

#### Fuzzy-set Qualitative Comparative Analysis (fsQCA)

Fuzzy-set qualitative comparative analysis (fsQCA) was conducted to examine the configurational pathways underlying the participants’ anxiety symptoms. This method enables multiple combinations of conditions associated with an outcome and is particularly suitable for capturing causal complexity and equifinality [16].

##### Calibration of variables

All the variables were calibrated into fuzzy-set membership scores ranging from 0 (full non-membership) to 1 (full membership). The outcome variable, anxiety symptoms, was calibrated using the direct method based on SCAS-S total scores (range: 0–57), with higher scores indicating greater anxiety severity.

Calibration was performed according to established guidelines [18] and informed by both normative evidence and clinical criteria. Studies based on Chinese normative samples have indicated that scores around the 90th percentile, which corresponds to values of approximately 38–42, reflect an increased risk of anxiety [18]. Clinically significant anxiety has been defined as a score exceeding 1.5 standard deviations above the mean, which corresponds to a value of approximately 35 in general pediatric populations.

For this study, three qualitative anchors were specified based on the above-described considerations. A threshold of ≥ 40 was set for full membership in the set of high anxiety symptoms; this represented clearly elevated and clinically meaningful anxiety. The crossover threshold was set at 35, indicating clinical significance and maximum ambiguity in set membership. Full non-membership was set at ≤ 20, which approximated the normative mean levels and indicated low anxiety. The set of low anxiety symptoms is defined here as the negation of the high anxiety set.

Continuous coping variables, namely acceptance, emotional reaction, distancing, wishful thinking, self-comfort, avoidance, and general coping self-assessment, were calibrated using the 75th, 50th, and 25th percentiles of each variable as the thresholds for full membership, crossover, and full non-membership, respectively. These calibrations represent relatively high and low levels of each coping strategy within the sample.

Age was calibrated to indicate a “relatively younger” status, while its negation indicated “older” children. Binary variables (gender, sibling status, insurance, and emotional expression) were treated as crisp sets (0 = absence, 1 = presence).

##### Analysis procedure

Separate analyses were conducted for the outcomes of high and low anxiety symptoms. The configurations were evaluated using consistency and coverage, which indicate the degree to which a configuration is associated with the outcome and its empirical relevance, respectively.

1. *Necessity analysis*
  We first assessed whether any single condition was a requisite for the outcome. In line with established standards, a consistency threshold of 0.90 was set to identify the necessary conditions.

(2) *Sufficiency analysis*
  Configurations of conditions were evaluated to determine consistency and coverage. Following prior recommendations [14], the configurations were retained if they met a consistency threshold of ≥ 0.75 and a raw coverage threshold of ≥ 0.05.

### Ethical considerations

This study was conducted in accordance with the ethical principles outlined in the Declaration of Helsinki (World Medical Association). The study protocol was approved by the Survey and Behavioural Research Ethics Committee of the Chinese University of Hong Kong (Approval No. P84709870). Assent to participate in the study was obtained from all the participating children, and written informed consent was obtained from their parents or legal guardians.

## Results

### Sample characteristics

A total of 390 children with hematological cancers were included in the study (Table 1). The mean age of the participants was 9.93 ± 2.52 years, and the mean anxiety symptom score was 36.01 ± 10.82. Of the participants, 228 were boys, and 162 were girls; 297 had siblings, and 93 were only children; and 383 had health insurance, while seven did not. Furthermore, 295 and 95 reported expressing and not expressing distress, respectively. Regarding diagnosis, acute lymphoblastic leukemia was the most common type (*n* = 281), followed by acute myeloid leukemia (*n* = 56), mixed diagnoses (*n* = 26), Hodgkin lymphoma (*n* = 19), and non-Hodgkin lymphoma (*n* = 8).

**Table 1:** Sample characteristics and descriptive statistics of study variables

Characteristic	All (*n* = 390)
Demographic characteristics	
Age (years), Mean ± SD	9.93 ± 2.52
Gender, n (%)	
Male	228(58.5%)
Female	161(41.3%)
Siblings, n (%)	
Have	297(76.15%)
One-child	93(23.85%)
Insurance	
Have	383 (98.21%)
Do not have	7 (1.79%)
Emotional disclosure	
Does not express emotions	94 (24.10%)
Express emotions	295 (75.64%)
Clinical characteristics	
Diagnosis, n (%)	
ALL	281(73.3%)
AML	56(10%)
HL	19(6.7%)
NHL	8(6.7%)
Mixed type	26(3.3%)
Outcomes (Mean ± SD)	
Anxiety symptoms	36.01 ± 10.82
Cope	
Accept	3.42 ± 1.04
Emotional reaction	2.37 ± 0.88
Distance	2.81 ± 1.01
Wishful thinking	4.82 ± 1.01
Comfort	3.53 ± 1.07
Avoid	2.95 ± 1.14
Global coping self-assessment	3.76 ± 1.08

Due to missing data for some variables, category totals may not equal 390, and percentages may not sum to 100%

**Fig. 1:**
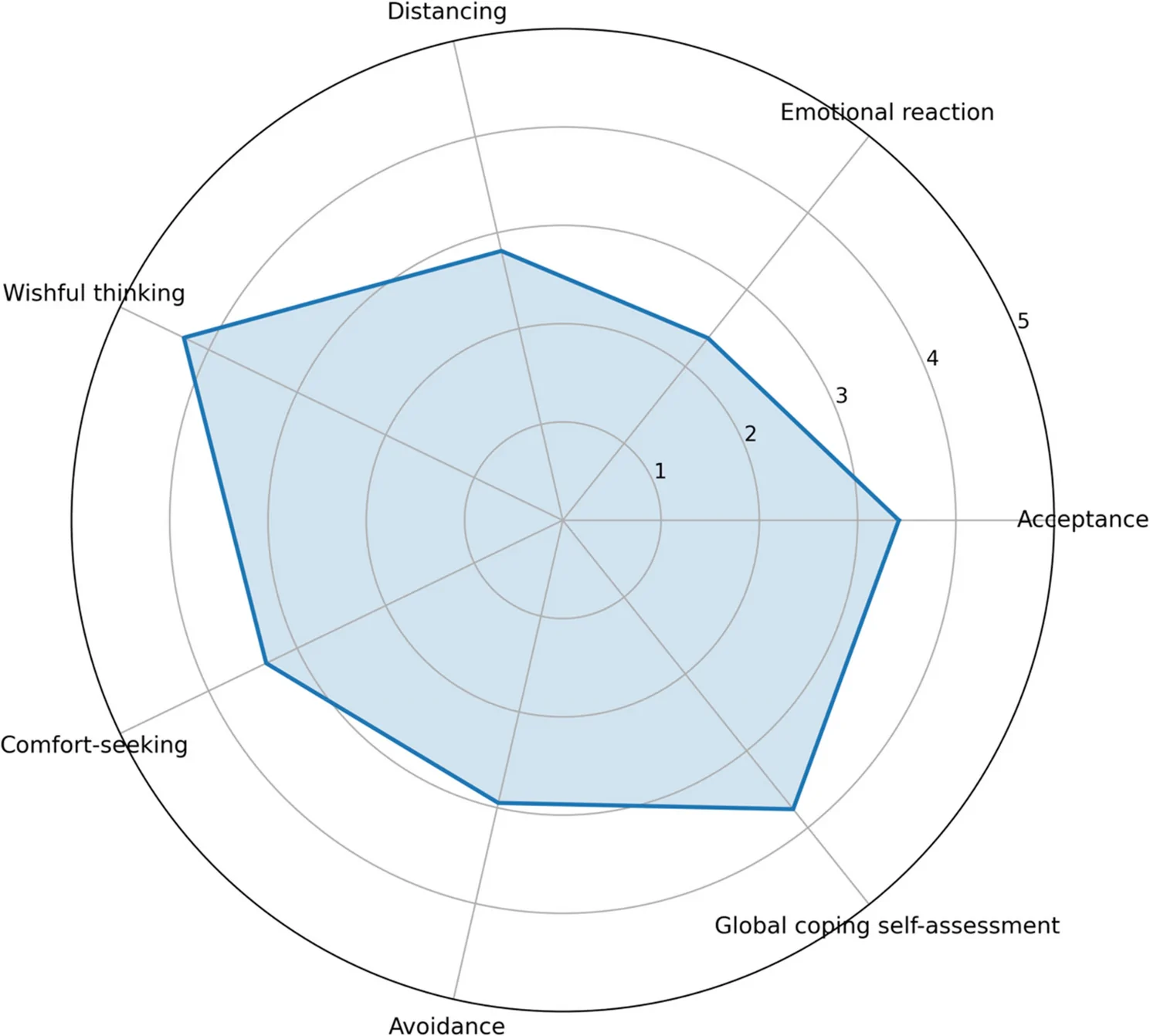
The radar plot of coping

The mean score for anxiety symptoms was 36.01 ± 10.82. The item‑level mean scores for coping strategies were as follows: acceptance, 3.42 ± 1.04; emotional reaction, 2.37 ± 0.88; distancing, 2.81 ± 1.01; wishful thinking, 4.28 ± 1.01; comfort‑seeking, 3.35 ± 1.07; avoidance, 2.95 ± 1.14; and overall coping self‑assessment (3.76 ± 1.08). As shown in the radar plot (Fig. 1), the highest mean score was for acceptance, followed by self-comfort and emotional reaction, whereas the lowest was for avoidance. The correlations ranged from − 0.28 to 0.67, suggesting that the coping dimensions were related but not redundant. The strongest correlation was observed between comfort coping and avoidance coping (*r* = 0.67), indicating moderate-to-strong overlap that should be considered when interpreting the regression results (supplementary Figure S1).

### Coping-related factors associated with anxiety symptoms

Multiple linear regression analysis identified coping-related variables as the main factors associated with anxiety symptoms (Table 2). The final model explained 47.1% of the variance in anxiety symptoms (R² = 0.471).

**Table 2:** Final multiple linear regression model predicting anxiety symptoms

Predictor	B	SE	95% CI	*p*
Intercept	32.34	4.3	23.89 to 40.79	< 0.001
Male (vs. female)	-1.83	0.84	-3.47 to -0.19	0.029^*^
Only child	0.9	0.96	-0.98 to 2.78	0.349
Insured	-2.37	3.08	-8.43 to 3.70	0.443
Expresses emotions	-0.52	1	-2.49 to 1.45	0.603
Age	-0.25	0.17	-0.58 to 0.08	0.132
Acceptance coping	-0.08	0.09	-0.27 to 0.10	0.373
Emotional reaction coping	1.17	0.09	0.99 to 1.35	< 0.001^**^
Distancing coping	0.01	0.13	-0.24 to 0.27	0.92
Wishful thinking coping	-0.26	0.18	-0.62 to 0.10	0.158
Self-comfort coping	0.35	0.13	0.09 to 0.60	0.008^*^
Avoidance coping	-0.13	0.17	-0.46 to 0.21	0.454
Overall coping	-1.78	0.45	-2.67 to -0.90	< 0.001^**^

*: *P*<0.05; ** *P*<0.001; *B*  Unstandardized coefficient, *SE*  Standard error, *CI*  Confidence interval. Model fit: R² = 0.471, adjusted R² = 0.454, F (12, 375) = 27.84, *p* < 0.001; *n* = 388

Emotional coping showed the strongest positive association with anxiety symptoms (β = 1.171, *p* < 0.001), indicating that greater emotional reactivity was associated with higher anxiety symptoms. Comfort coping was also positively associated with anxiety symptoms (β = 0.348, *p* = 0.008). In contrast, the global coping self-assessment score was negatively associated with anxiety symptoms (β = −1.780, *p* < 0.001), suggesting that children with higher perceived coping ability reported lower anxiety symptoms. Male sex was associated with lower anxiety symptoms (β = −1.827, *p* = 0.029), whereas no other demographic variables showed significant associations with anxiety symptoms.

### Configurational patterns of anxiety symptoms (fsQCA)

The fsQCA identified multiple configurations associated with both high and low anxiety symptoms, indicating configurational complexity and equifinality. Detailed configurations are presented in Table 3.

**Table 3:** Representative configurations associated with high and low anxiety symptoms (fsQCA)

Outcome	Configuration	Consistency	Raw Coverage	Unique Coverage
High anxiety symptoms	high_cope_distance * low_cope_accept * low_global coping self-assessment * not_express	0.983	0.064	0.038
	high_cope_avoid * high_cope_distance * low_cope_accept * not_express	0.982	0.052	0.031
	high_cope_emotion * high_cope_distance * low_cope_accept * not_express	0.981	0.063	0.038
	high_cope_emotion * low_cope_distance * female * only_child	0.979	0.054	0.032
	high_cope_distance * high_cope_comfort * low_cope_accept * not_express	0.976	0.05	0.03
Low anxiety symptoms	high_global coping self-assessment * low_cope_emotion * low_cope_avoid * older_age	0.903	0.053	0.032
	high_cope_accept * low_cope_emotion * low_cope_avoid * older_age	0.889	0.058	0.035
	high_global coping self-assessment * low_cope_emotion * low_cope_comfort * older_age	0.864	0.051	0.031
	high_cope_accept * high_global coping self-assessment * low_cope_emotion * low_cope_comfort	0.859	0.212	0.127
	high_global coping self-assessment * low_cope_emotion * low_cope_comfort * express emotion	0.858	0.237	0.142

Only representative configurations with high consistency are presented; * indicates logical; “Older age” refers to relatively older children within the sample

#### Configurations associated with high anxiety symptoms

**Fig. 2:**
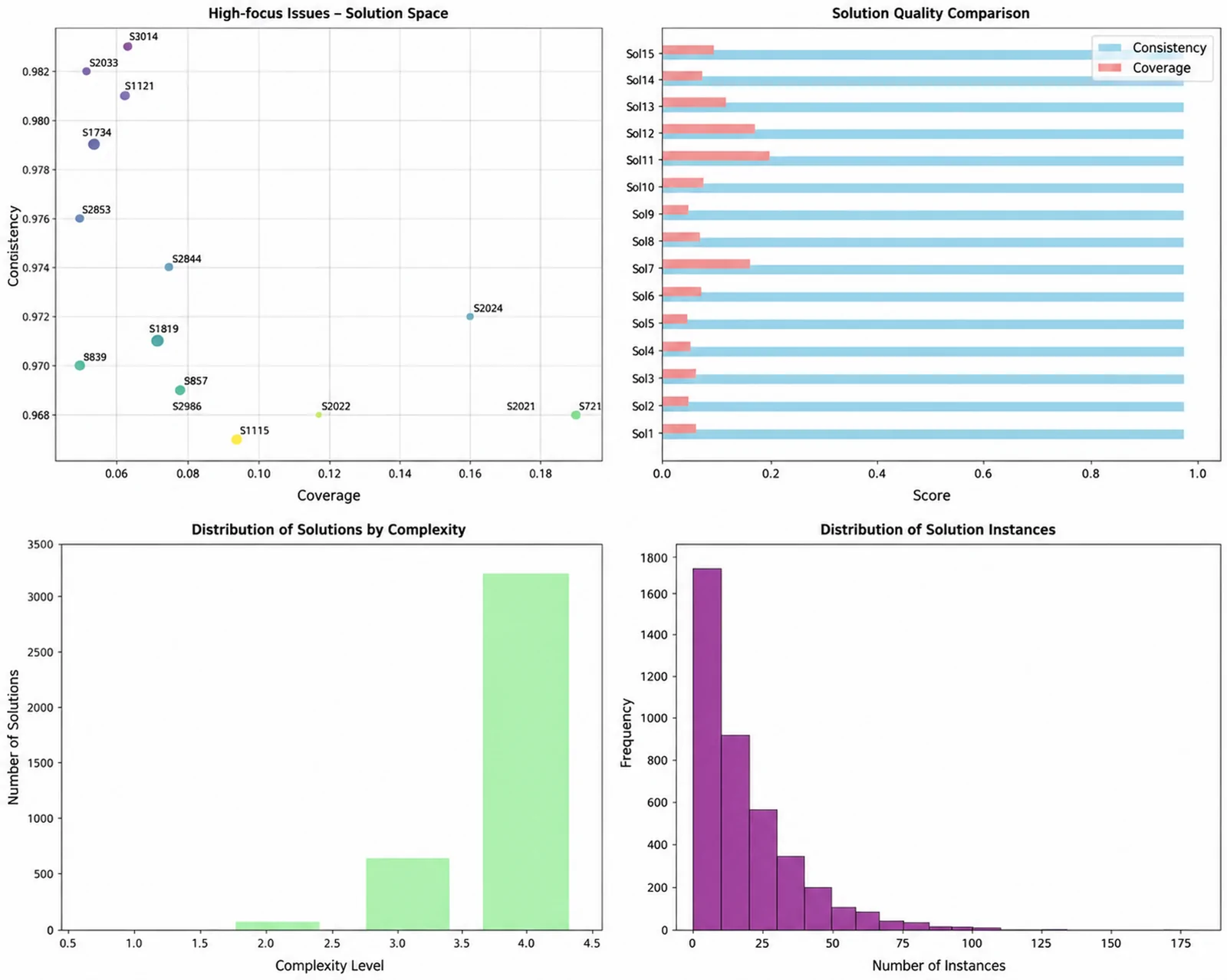
High anxiety symptoms pathways

As shown in Fig. 2, high anxiety symptoms were characterized by numerous configurations with high consistency values, generally > 0.96, but relatively low coverage values, suggesting heterogeneous patterns observed in specific subsets of participants rather than dominant pathways for the whole sample.

The configuration with the highest consistency was high distancing coping AND low acceptance coping AND low global coping self-assessment AND limited emotional expression (consistency = 0.983, raw coverage = 0.064). This configuration indicates that high anxiety symptoms were associated with the co-occurrence of maladaptive coping, insufficient adaptive coping resources, and limited emotional expression.

As shown in Table 3, several recurring patterns were observed. Low acceptance coping appeared frequently, indicating lower use of adaptive coping resources. Maladaptive coping components, particularly emotional reactivity, distancing, and avoidance, were common across configurations. Reduced emotional expression also emerged repeatedly, suggesting that limited emotional expression may be an important feature of some high-anxiety configurations when combined with less adaptive coping patterns.

#### Configurations associated with low anxiety symptoms

**Fig. 3:**
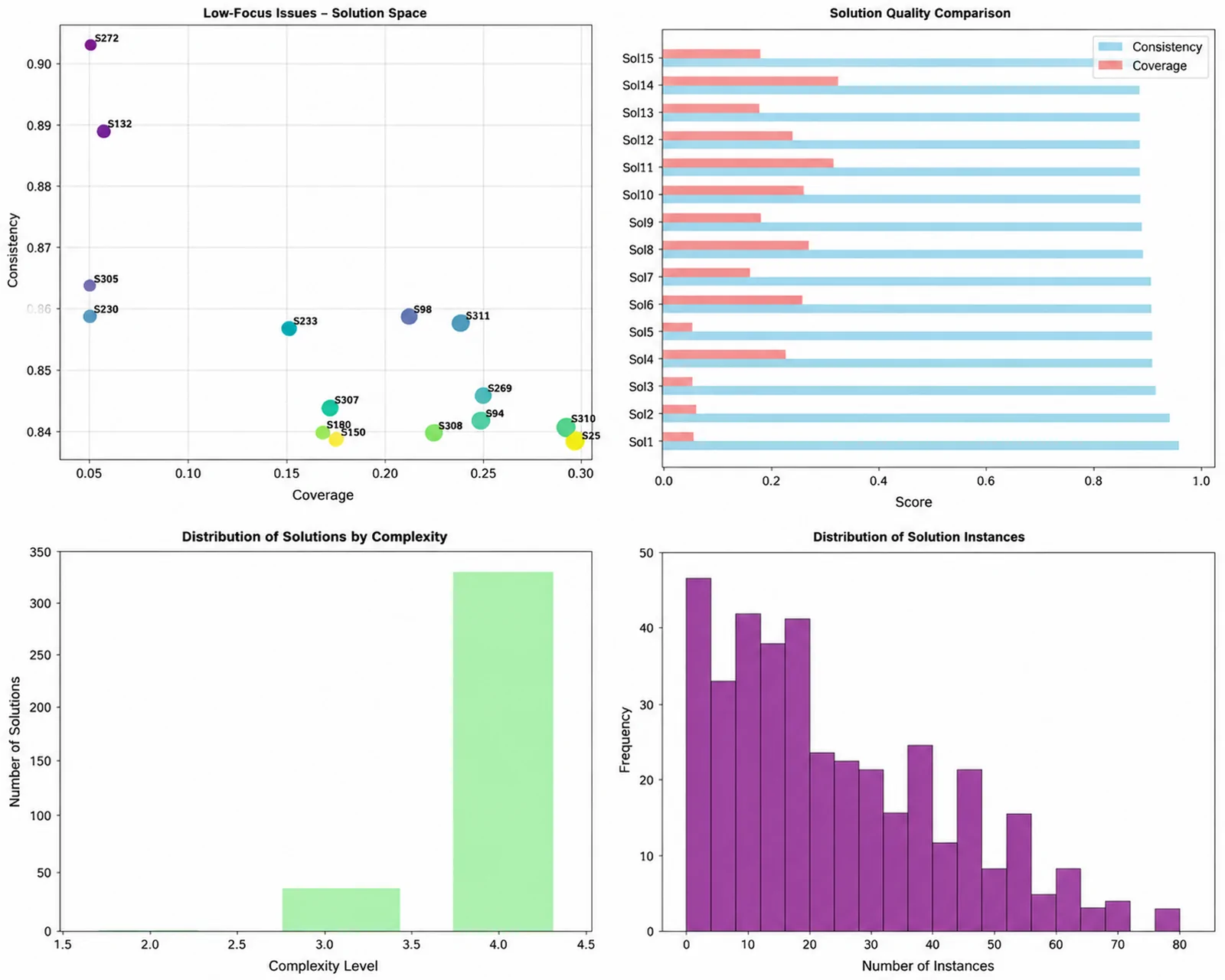
Low anxiety symptoms pathways

As illustrated in Fig. 3, the configurations associated with low anxiety symptoms met the consistency criteria but should not be interpreted as more consistent than the high-anxiety configurations. The configuration with the highest consistency was high global coping self-assessment, low emotional reaction coping, low avoidance coping, and older age (consistency = 0.903, raw coverage = 0.053). This configuration suggests that lower anxiety symptoms were associated with stronger global coping self-assessment, reduced emotional reactivity, and less reliance on avoidance strategies, particularly among relatively older children.

Low emotional reaction coping emerged repeatedly across low-anxiety configurations, often in combination with higher levels of adaptive coping, such as acceptance coping. Reduced avoidance was also frequently observed.

Although the raw coverage of the highest-consistency configuration was relatively low, fsQCA identifies sufficient configurations rather than dominant patterns for the entire sample. Therefore, this configuration should be interpreted as a specific pattern observed in a subset of participants, not as the most common pathway.

#### Necessity analysis

No single condition reached the threshold for strict necessity (consistency > 0.90). However, emotional coping showed the highest consistency across outcomes, indicating that it may be an important condition associated with anxiety symptoms, although it was not necessary or sufficient on its own.

#### Summary of configurational findings

Across the analyses, coping characteristics emerged as central factors associated with anxiety symptoms. Emotional coping was consistently associated with higher anxiety symptoms, whereas higher global coping self-assessment was associated with lower anxiety symptoms. Configurational analysis further suggested that anxiety symptoms were associated with combinations of coping characteristics rather than single factors alone, with distinct configurations for high and low anxiety symptoms. Importantly, the finding that the configurations associated with high and low anxiety symptoms were not simple mirror opposites suggests configurational asymmetry between patterns associated with higher and lower anxiety symptoms.

## Discussion

In this multicenter study of children with hematological cancers, coping characteristics emerged as salient factors associated with anxiety symptoms. Across the analytic approaches, emotional reaction coping was consistently associated with higher anxiety symptoms, whereas higher global coping self-assessment was associated with lower anxiety symptoms. The configurational analysis further suggested that anxiety symptoms were associated not only with individual coping characteristics but also with combinations of coping-related conditions. Children with higher anxiety symptoms often showed a pattern of strong emotional reactions, together with limited coping resources and less emotional expression. In contrast, lower anxiety symptoms were more often observed in children who reported greater coping capacity and lower emotional reactivity. Taken together, these findings suggest that anxiety symptoms in this population are related to how children manage treatment-related stress, rather than being attributable to any single coping response.

### Interpretation in relation to the literature

Our findings are consistent with current evidence indicating that psychological distress remains prevalent among pediatric cancer patients, particularly those actively undergoing treatment. A recent systematic review and meta-analysis reported substantial levels of anxiety and related symptoms across the cancer trajectory, highlighting the importance of early identification of modifiable psychosocial factors [3].

Within this framework, coping is a crucial process for understanding psychological adjustment in response to illness-related stress [5]. The results of the current study suggest that heightened emotional reactivity was associated with higher anxiety symptoms. Unlike strategies targeting active regulation, emotional reaction coping encompasses responses such as acute worry, distress, and physiological arousal. In clinical practice, such reactions may be observed during procedures, such as venipuncture or lumbar puncture, during periods of physical discomfort, or when children anticipate treatment-related pain or uncertainty. When these reactions are intense or insufficiently regulated, they may be associated with a stronger perceived threat in clinical environments. In turn, the child’s capacity to navigate repeated and largely uncontrollable treatment stressors may be challenged, which could be related to persistent anxiety symptoms [21].

The observed negative association between global coping self-assessment and anxiety symptoms suggests that children with greater perceived coping capacity reported lower anxiety symptoms. This finding may indicate the importance of perceived coping confidence and flexible use of coping strategies during repeated treatment-related stressors. Children with higher global coping self-assessment may be better able to adjust their responses across different clinical situations, such as painful procedures, treatment uncertainty, or chemotherapy-related discomfort [22].

### Added value of the configurational approach

An important contribution of this study is the combined use of regression analysis and fsQCA. Regression analysis identified coping dimensions independently associated with anxiety symptoms, whereas fsQCA explored how coping characteristics co-occurred in relation to high and low anxiety symptoms. Therefore, the two approaches provided complementary information: regression described variable-level associations, while fsQCA described configurational patterns.

The fsQCA findings should be interpreted cautiously because the raw coverage values of individual configurations were relatively low. These configurations therefore represent specific subsets of participants rather than dominant pathways for the entire sample. Nevertheless, they provide useful exploratory insight into the heterogeneity of coping profiles associated with anxiety symptoms. This approach extends previous pediatric oncology research, which has largely relied on variable-centered methods to examine the independent associations of individual coping dimensions with psychological outcomes [22]. By incorporating fsQCA, the present study also explored how different combinations of coping characteristics were associated with anxiety symptoms, providing complementary information beyond the regression analysis.

High anxiety symptom configurations appeared more heterogeneous in this study, whereas fewer configurations were identified for low anxiety symptoms. However, because some high-anxiety configurations showed higher consistency values than low-anxiety configurations, the findings should not be interpreted as evidence that low-anxiety configurations were more consistent. The fact that configurations associated with high and low anxiety symptoms were not simple mirror opposites suggests configurational asymmetry. This means that the combinations of conditions associated with higher anxiety symptoms may differ from those associated with lower anxiety symptoms. Clinically, this distinction may be useful because children with similar anxiety symptom levels may present different coping profiles and may therefore require different forms of psychosocial support [11, 23]. 

### Clinical and nursing implications

The study findings have important implications for pediatric oncology nursing practice. Given that coping processes may operate in combination rather than in isolation, psychosocial assessments should move beyond single indicators toward a more comprehensive evaluation of coping profiles. Patterns such as heightened emotional reactivity, reduced adaptive coping capacity, and limited emotional expression may help nurses identify children with higher anxiety symptoms or greater need for psychological support.

From a clinical perspective, these results support more individualized psychosocial care. Rather than applying uniform interventions, nursing care could be tailored according to children’s coping profiles. For example, children with high emotional reactivity may benefit from anticipatory guidance before invasive procedures, breathing exercises, relaxation training, distraction techniques, and age-appropriate emotion-regulation strategies. Children with limited coping resources may require structured coping-skills support, including preparation for treatment procedures, problem-solving guidance, and positive reinforcement of successful coping attempts. Children who avoid expressing distress may benefit from supportive communication, play-based approaches, drawing, storytelling, or other developmentally appropriate methods to facilitate emotional expression.

The findings also highlight the importance of early identification. As frontline care providers with continuous patient contact, nurses are well-positioned to observe less adaptive coping patterns during routine care. Integrating brief assessments of coping and emotional responses into nursing practice may facilitate timely psychological referral and supportive care for children with higher anxiety symptoms.

Finally, interventions should aim not only to relieve distress but also to strengthen children’s adaptive coping capacity. Approaches that promote coping flexibility, support emotional processing, and encourage appropriate emotional expression may be particularly relevant in pediatric oncology settings, where children face ongoing and unpredictable stressors.

### Contribution to current knowledge

This study contributes to the literature by showing that coping characteristics are associated with anxiety symptoms both at the individual-variable level and within broader configurations. Rather than repeating the clinical implications above, this section emphasizes the conceptual and methodological contribution of the study. Regression analysis identified the relative importance of specific coping dimensions, particularly emotional reaction coping and global coping self-assessment, whereas fsQCA identified heterogeneous configurations associated with similar anxiety symptom levels.

The findings support a more holistic conceptualization of psychological adjustment in children with cancer by showing that anxiety symptoms may be associated with multiple co-occurring coping characteristics. The study also highlights configurational asymmetry between patterns associated with high and low anxiety symptoms. High anxiety symptoms were associated with more diverse configurations, whereas fewer configurations were identified for low anxiety symptoms. These findings should be interpreted as exploratory and descriptive rather than causal, but they may help refine future theoretical models of psychosocial adjustment in pediatric oncology.

### Limitations

Several limitations of this study should be acknowledged. First, the use of self-report measures may have introduced response bias, particularly among younger children. Second, although the multicenter design is a strength, the sample was restricted to children with hematological cancers who were undergoing chemotherapy. Therefore, the findings may not be generalizable to children with solid tumors, childhood cancer survivors, or patients receiving other forms of treatment or at different stages of the cancer trajectory. Third, the fsQCA approach is exploratory and partly dependent on calibration decisions, although these were specified a priori and applied consistently. Fourth, treatment stage was not collected in the present study. Because children at different phases of chemotherapy may experience different levels of symptom burden, uncertainty, and emotional distress, future studies should examine whether the associations between coping characteristics and anxiety symptoms differ across treatment stages. Finally, potentially relevant family- and treatment-level variables, such as caregiver distress and treatment intensity, were not assessed. Future longitudinal and multicenter studies involving broader pediatric oncology populations are needed to clarify the directionality of the observed associations, examine whether the identified coping configurations are consistent across different cancer types and treatment stages, and evaluate whether coping-informed nursing interventions are associated with sustained reductions in anxiety symptoms over time.

## Conclusions

The results of this multicenter study indicate that anxiety symptoms in children with haematological cancers are closely associated with individual coping dimensions and broader coping-related configurations. High anxiety symptoms were associated with coping profiles characterized by maladaptive responses, limited adaptive coping resources, and reduced emotional expression. In contrast, low anxiety symptoms were associated with more adaptive and balanced coping patterns. By combining regression and fsQCA, this study provides both variable-level and configuration-level evidence showing that anxiety risk may arise through different coping-related patterns. These findings support the importance of assessing children’s coping characteristics in pediatric oncology nursing practice, which may help nurses identify children at increased risk of anxiety earlier. Nursing care should not only aim to reduce distress but also strengthen children’s adaptive coping capacity through individualized psychosocial support during treatment.

## Supplementary Information

Supplementary Material 1.

## Data Availability

The datasets used and analysed in the current study are available from the corresponding authors upon reasonable request.
