# Supplementary material for: Association of coping pathways with anxiety symptoms in children with hematological cancers: a cross-sectional study using regression and fuzzy-set qualitative comparative analysis
Source: BMC Psychol. 2026 Jul 27;14:1525. doi: 10.1186/s40359-026-05216-8 (PMC13689067; doi:10.1186/s40359-026-05216-8)
Supplement: Supplementary file 1 [file 40359_2026_5216_MOESM1_ESM.docx]

Supplementary Figure S1. Pearson correlation matrix of coping dimensions and anxiety symptoms.
